# Angiotensin-Converting Enzyme 2 (ACE2) Signaling in Pulmonary Arterial Hypertension: Underpinning Mechanisms and Potential Targeting Strategies

**DOI:** 10.3390/ijms242417441

**Published:** 2023-12-13

**Authors:** Kostas A. Papavassiliou, Vassiliki A. Gogou, Athanasios G. Papavassiliou

**Affiliations:** 1 First University Department of Respiratory Medicine, ‘Sotiria’ Hospital, Medical School, National and Kapodistrian University of Athens, 11527 Athens, Greece; gogvanessa@gmail.com; 2 Department of Biological Chemistry, Medical School, National and Kapodistrian University of Athens, 11527 Athens, Greece

**Keywords:** angiotensin-converting enzyme 2, angiotensin-(1-7), pulmonary arterial hypertension

## Abstract

Pulmonary arterial hypertension (PAH) is a debilitating progressive disease characterized by excessive pulmonary vasoconstriction and abnormal vascular remodeling processes that lead to right-ventricular heart failure and, ultimately, death. Although our understanding of its pathophysiology has advanced and several treatment modalities are currently available for the management of PAH patients, none are curative and the prognosis remains poor. Therefore, further research is required to decipher the molecular mechanisms associated with PAH. Angiotensin-converting enzyme 2 (ACE2) plays an important role through its vasoprotective functions in cardiopulmonary homeostasis, and accumulating preclinical and clinical evidence shows that the upregulation of the ACE2/Angiotensin-(1-7)/MAS1 proto-oncogene, G protein-coupled receptor (Mas 1 receptor) signaling axis is implicated in the pathophysiology of PAH. Herein, we highlight the molecular mechanisms of ACE2 signaling in PAH and discuss its potential as a therapeutic target.

## 1. Introduction

Pulmonary arterial hypertension (PAH) is a devastating disorder characterized by an increase in pulmonary vascular resistance which results in right-ventricular heart overload and failure. It represents one of five groups of pulmonary hypertension and, according to the 2022 European Society of Cardiology (ESC)/European Respiratory Society (ERS) guidelines, is now hemodynamically defined by right-heart catheterization demonstrating a mean pulmonary artery pressure >20 mm Hg at rest, a pulmonary vascular resistance >2 Wood units, and pulmonary artery wedge pressure ≤15 mm Hg [1]. Based on underlying etiology, PAH is further divided into subgroups (idiopathic, heritable, drug- and toxin-induced PAH, PAH associated with connective tissue disease, HIV infection, congenital heart disease, portal hypertension, and schistosomiasis) [1]. Patients with PAH initially present with nonspecific symptoms and typically display breathlessness (dyspnea) on exertion and fatigue, whereas in advanced disease chest pain, fainting, and heart failure symptoms are common. Despite a lack of knowledge of the exact causes behind the development of PAH, advances in research have provided a better understanding of the underlying pathophysiology and, most importantly, have led to the development and clinical use of several effective drug therapies. Current therapy approved for patients with PAH includes drugs that upregulate the nitric oxide-cyclic guanosine monophosphate signaling pathway (e.g., tadalafil, sildenafil, riociguat), antagonists that block the endothelin signaling pathway (e.g., ambrisentan, bosentan), and agonists that stimulate the prostacyclin signaling pathway (e.g., treprostinil, epoprostenol). Untreated, PAH patients have a dismal prognosis typically progressing to right-heart failure and ultimately death. Conversely, with the use of currently approved therapies specific for PAH, there has been a substantial improvement in the 5-year survival of PAH patients reaching more than 60% compared to 34% in 1991 when effective drugs were not available [2]. As first-line therapy, clinicians now administer drug combinations which target multiple signaling pathways associated with PAH, and this therapeutic strategy has improved PAH survival rates [2].

Advances in PAH research demonstrate that PAH is a complex, multifactorial pathological condition involving a plethora of signaling pathways and different types of cells, such as endothelial cells, smooth muscle cells, fibroblasts, and inflammatory cells. The pulmonary arteries of patients with PAH become narrowed due to abnormal vascular remodeling processes affecting all three layers of the vessel wall. Specifically, endothelial-mediated vasoconstriction, cell proliferation, and fibrosis occur in the intima layer; hypertrophy, smooth muscle cell hyperplasia, and vasoconstriction occur in the media layer; and increased inflammation as well as fibrosis-induced vascular stiffening occur in the adventitia layer of the pulmonary artery wall [3,4]. At the molecular level, multiple mechanisms have been implicated in PAH and may interact with each other, including nitric oxide, prostacyclin, endothelin-1, serotonin, endothelial dysfunction, calcium and potassium ion channels, mitochondrial metabolic dysfunction, inflammatory cytokines, sex hormones (estrogen), renin–angiotensin–aldosterone system (RAAS), bone morphogenic protein receptor 2 (BMPR2) mutations, and epigenetic alterations (DNA methylation, histone acetylation, microRNAs) [5].

Here, we focus on the role of angiotensin-converting enzyme 2 (ACE2) signaling in the pathophysiology of PAH, probing its molecular underpinnings and evaluating its clinical potential as a therapeutic target.

## 2. ACE2 Signaling in PAH Pathophysiology—Molecular Mechanisms

**Figure 1:**
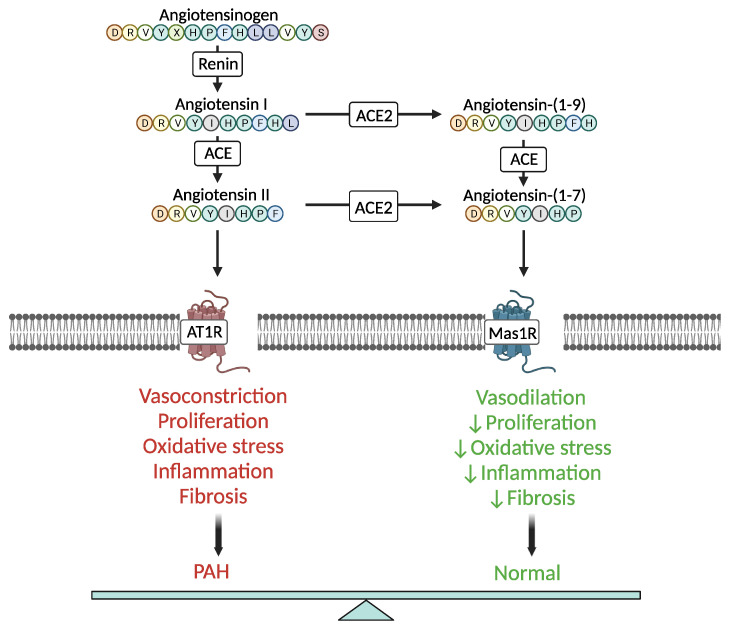
Simplified schematic illustration of the two counterbalancing axes (ACE2/Ang-(1-7)/Mas 1 receptor and ACE/Ang II/AT1R) of the renin–angiotensin system in PAH pathophysiology. ACE, angiotensin-converting enzyme; ACE2, angiotensin-converting enzyme 2; AT1R, angiotensin II type 1 receptor; Mas1R, Mas 1 receptor; PAH, pulmonary arterial hypertension. This figure was created using the tools provided by BioRender.com (accessed on 10 December 2023).

Evidence shows that a dysregulated RAAS contributes to the development and progression of PAH [6,7,8]. The RAAS is divided into two signaling axes with opposing and coordinating functions, namely, the ACE/Angiotensin II (Ang II)/Angiotensin II type 1 receptor (AT_1_R) axis, and the ACE2/Ang-(1-7)/Mas 1 receptor axis [9,10]. The ACE/Ang II/AT_1_R axis is upregulated in PAH and has been linked to the deleterious cardiopulmonary effects observed in PAH, promoting aberrant proliferation, migration, resistance to apoptosis, vasoconstriction, hypertrophy, inflammation, fibrosis, and oxidative stress, all of which enhance the abnormal vascular remodeling process in the pulmonary artery [6,11,12,13,14,15,16,17]. The harmful effects of the ACE/Ang II/AT_1_R signaling pathway in the cardiopulmonary system are counterbalanced by the vasoprotective ACE2/Ang-(1-7)/Mas 1 receptor signaling pathway. ACE2 is mainly expressed in the lungs, heart, and kidneys. It is an ACE homologue but differs from the latter in terms of substrate specificity and function. ACE2 functions as a carboxymonopeptidase, catalyzing the conversion of Ang I and II into the biologically active peptides Ang-(1-9) and Ang-(1-7), respectively. In turn, Ang-(1-7), the main product of ACE2 binds to the Mas 1 receptor, a G protein-coupled receptor, eliciting downstream processes (Figure 1) [18,19,20].

Activation of ACE2/Ang-(1-7)/Mas 1 receptor signaling leads to vasodilation and the inhibition of processes that contribute to anomalous vascular remodeling, such as proliferation, apoptosis, inflammation, fibrosis, hypertrophy, and oxidative stress [21]. The molecular mechanisms mediating these processes in PAH are summarized in Table 1. Mechanistically, ACE2 blocks the proliferation and migration of smooth muscle cells in the pulmonary artery by inhibiting their extracellular signal-regulated kinase 1/2 (ERK1/2)- and Janus kinase 2/signal transducer and activating transcription 3 (JAK2/STAT3)-mediated survival signaling [22]. ACE2/Ang-(1-7)/Mas 1 receptor signaling reduces inflammation and oxidative stress via downregulating the transcription of proinflammatory cytokines (IL-6, IL-1β, TNF-α) and gp91phox, a subunit of nicotinamide adenine dinucleotide phosphate (NADPH) oxidase, respectively, while upregulating the anti-inflammatory cytokine IL-10 [21]. Furthermore, the ACE2/Ang-(1-7)/Mas 1 receptor pathway counteracts the fibrotic effects present in PAH by downregualting the mRNA levels of transforming growth factor-β and the production of collagen fibers [21]. Microvesicles derived from mesenchymal stem cells relieved the pulmonary artery pressure, right-ventricular hypertrophy index, pulmonary vessel wall thickness index, pulmonary vessel lumen area index, the inflammation score, and the collagen fiber volume fraction. Furthermore, the mRNA expression of ACE2 in the lung tissues and the levels of Ang-(1-7) in the plasma were both upregulated, while ACE and Ang-II levels were downregulated in the microvesicle group compared with the PAH group. These protective effects observed in the microvesicle group were abolished after administration of A-779, which is a Mas receptor inhibitor, suggesting that the ACE2/Ang-(1-7)/Mas 1 receptor pathway is responsible for these positive effects in the cardiopulmonary system [23]. Activation of ACE2 by the chemical compound resorcinolnaphthalein improved the pulmonary artery endothelial function in rat PAH models via inducing the release of nitric oxide due to ACE2-mediated phosphorylation of endothelial nitric oxide synthetase [24]. With the use of a rat model which overexpressed Ang-(1-7), Gomes et al. [25] evaluated the potential downstream pathways associated with Ang-(1-7)-mediated cardioprotection in PAH and found that Ang-(1-7) utilizes a nitric oxide/guanosine 3′,5′-cyclic monophosphate-dependent pathway to prevent cardiomyocyte hypertrophy [25]. In a pneumonectomy and monocrotaline-induced PAH rat model, the protein levels of anti-apoptotic protein Bcl-2 and the Hippo signaling pathway effector yes-associated protein (YAP) were both increased, whereas those of the pro-apoptotic proteins caspase-3 and Bax were reduced. Activation of ACE2 with resorcinolnaphthalein promoted pulmonary arterial cell apoptosis via Hippo/YAP signaling, resulting in the suppression of PAH-related vascular remodeling [26]. In a study where ACE2 was overexpressed in rat primary pulmonary arterial smooth muscle cells, their proliferation and migration were significantly inhibited and the development of hypoxia-induced pulmonary hypertension was prevented [27]. In another PAH rat model induced by monocrotaline and pneumonectomy, administration of resorcinolnaphthalein improved hemodynamics, pathological changes, and endothelial dysfunction, all of which were associated with an increase in the levels of ACE2 and Ang-(1-7) and a decrease in the levels of ACE and Ang II. All the beneficial effects of ACE2 activation were inhibited by the Mas 1 receptor antagonist A-779 [28]. Zhang et al. found that exposure of rat lung to chronic hypoxia stimulated the hypoxia-inducible factor 1–dependent expression of the microRNA let-7b, which, in turn, led to the downregulation of ACE2 expression. Further data revealed that let-7b induced the proliferation and migration of pulmonary artery smooth muscle cells, and knock-out of let-7b in a hypoxia-induced pulmonary hypertension rat model mitigated right-ventricular heart hypertrophy and abnormal vascular remodeling by restoring ACE2 expression [29]. Using a monocrotaline- and hypoxia-induced rat model of PAH, Wang et al. showed that ACE2 inhibits the expression of focal adhesion kinase (FAK) and its downstream effectors, and promotes the expression of pro-apoptotic caspase-3. Additionally, histopathological analysis demonstrated that ACE2 activation via diminazene (DIZE) downregulated FAK expression in pulmonary arterioles and promoted pulmonary artery smooth muscle cell apoptosis, leading to the alleviation of PAH [30]. Analysis of blood samples from PAH patients reveal low ACE2 levels; however, the underlying mechanisms remain unknown. A recent study by Zhang et al. identified an interesting mechanism related to the stability of ACE2. According to the in vitro and in vivo data of the study, AMP-activated protein kinase (AMPK) phosphorylates ACE2 at its Ser680 residue promoting its stability and stimulating an increase in Ang-(1-7) and nitric oxide levels. The authors also provided data showing low levels of phosphorylated AMPK, phosphorylated ACE2, and ACE2 in lung biopsies derived from patients with idiopathic PAH, validating in this way the clinical relevance of AMPK-dependent ACE2 phosphorylation [31]. Along these lines, Shen et al. recently revealed that murine double minute 2 (MDM2)-mediated ubiquitination of ACE2 at the K788 residue triggers the degradation of ACE2 and contributes to the development of PAH [32]. Another potential mechanism which may explain the decreased ACE2 levels and activity in PAH could be the presence of autoantibodies against ACE2 especially in patients with connective tissue diseases [33,34]. Chronic cigarette exposure may also decrease the protein levels of ACE2, as reported by Yuan et al. who investigated the effects of smoking on the RAAS in a rat model of smoking-induced PAH [35].

## 3. Clinical Relevance of ACE2 Signaling in PAH—Potential as Therapeutic Target

Based on the above findings, the ACE2/Ang-(1-7)/Mas 1 receptor signaling pathway represents a promising new target for the treatment of PAH. Several preclinical studies highlight the potential of ACE2 as a therapeutic target in PAH, and a few clinical trials have already provided the grounds for realizing this potential.

Shenoy and colleagues evaluated the pharmacological actions of diminazene aceturate (DIZE) a compound which enhances the enzymatic activity of ACE2 in experimental models of PH. Treatment with DIZE significantly prevented the development of PH in all of the animal models studied [36]. The protective effects were associated with upregulation of ACE2/Ang-(1-7)/Mas 1 receptor signaling, decreased proinflammatory cytokine levels, improved pulmonary vasoreactivity, and improved heart function, while blocking ACE2 with C-16 obliterated these beneficial effects. Initiation of DIZE treatment in rat PH models halted disease progression. Additionally, the authors reported that DIZE improved angiogenic progenitor cell function [36]. Another group of investigators also used DIZE in a rat PAH model generated via left pneumonectomy combined with vascular endothelial growth factor inhibition. DIZE treatment significantly improved hemodynamics by reducing the mean pulmonary artery pressure, reversing the pathological pulmonary vascular remodeling, and decreasing brain natriuretic peptide (BNP) levels within the right ventricle and in the plasma. Additionally, ACE expression was downregulated, while Ang-(1-7) and endothelial nitric oxide synthetase expression were upregulated [37].

Resorcinolnaphthalein is another ACE2 activator which has shown promising results in PAH preclinical studies. Specifically, activation of ACE2 with resorcinolnaphthalein improves endothelial function, reduces neointimal occlusive lesions in pulmonary arteries, downregulates the transcription of proinflammatory cytokines (tumor necrosis factor (TNF)-α, monocyte chemoattractant protein-1 (MCP-1), interleukin (IL)-6), upregulates the transcription of the anti-inflammatory cytokine IL-10, and suppresses pulmonary vascular remodeling by promoting the apoptosis of pulmonary arterial cells through the Hippo/YAP signaling pathway [26,28,38]. Additional studies have also identified several natural compounds, such as Tsantan Sumtang and magnolol, which are able to upregulate the expression of ACE2 and ameliorate pathological processes in different murine PAH models [39,40].

Shenoy and colleagues have developed a system that generates human ACE2 and Ang-(1-7) proteins within plant chloroplasts using transplastomic technology. A rat PAH model feeding on these bioengineered plants, demonstrated substantial cardiopulmonary improvements in experimental protocols for preventing and reversing PAH. Interestingly, the combination of ACE2 and Ang-(1-7) in the reversal protocol exhibited better results than single therapy [41,42]. In a similar fashion, lettuce chloroplasts were bioengineered by Daniell et al. to express ACE2 and Ang-(1-7), and when fed to monocrotaline-induced PAH rats, the development of PAH was attenuated [43].

Other therapeutic modalities at the preclinical stage that modulate the ACE2/Ang-(1-7)/Mas 1 receptor signaling pathway include ACE2 or Ang-(1-7) expression based on genetic-engineering methods, several activators of ACE2, such as Ang-(1-7), cyclic analogue of Ang-(1-7), XNT, NCP-2454, as well as Mas 1 receptor agonists (e.g., AVE0991) [21,36,44,45,46,47].

The innovative clinical study by Hemnes et al. used a recombinant form of human ACE2 (GSK2586881) and showed that infusion of a single dose is well-tolerated and effective in PAH patients as it improves cardiac output and pulmonary vascular resistance [48]. Simon et al. conducted a phase IIa clinical trial using larger doses of GSK2586881 and assessed cardiopulmonary hemodynamics in PAH patients who were already under treatment with approved therapies [49]. GSK2586881 failed to show any beneficial effects in mean pulmonary artery pressure, pulmonary vascular resistance, and cardiac index. Notwithstanding, GSK2586881 treatment led to a decrease in Ang II levels and an increase in Ang-(1-7) and Ang-(1-5) levels. Although there was a lack of short term benefits in terms of vasodilation in PAH patients, it will be interesting to evaluate the potential long term benefits of GSK2586881 associated with vascular remodeling [49].

## 4. Concluding Remarks—Challenges and Opportunities

**Figure 2:**
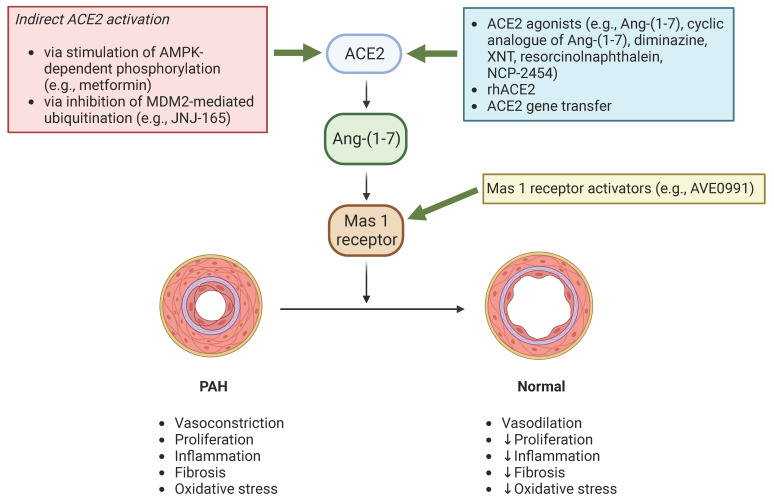
ACE2 signaling in PAH and potential therapeutic targeting. ACE2, angiotensin-converting enzyme 2; AMPK, AMP-activated protein kinase; Ang-(1-7), angiotensin-(1-7); Mas 1 receptor, MAS1 proto-oncogene, G protein-coupled receptor; MDM2, murine double minute 2; PAH, pulmonary arterial hypertension; rhACE2, recombinant human angiotensin-converting enzyme 2. This figure was created using the tools provided by BioRender.com (accessed on 10 December 2023).

Given the limited efficacy of currently available treatments for PAH, the development of novel therapeutics is of utmost importance. As discussed above, ACE2 exerts a multitude of beneficial effects on the cardiopulmonary system, resulting in the prevention and reversal of PAH. Therefore, genetically engineered approaches which upregulate ACE2 expression or activity and/or pharmacological ACE2 activators and recombinant human ACE2 may represent a novel therapeutic strategy in the management of PAH patients (Figure 2).

Nevertheless, there is a plethora of issues that must be resolved related to the molecular mechanisms through which ACE2 produces its beneficial effects in PAH. Addressing these issues will be vital in the challenging process of translating preclinical findings regarding ACE2 to clinical therapies for PAH. Firstly, we need to understand whether all ACE2 beneficial effects for the cardiopulmonary system emanate from its conversion of Ang II to Ang-(1-7). It may be the case that the enzymatic action of ACE2 on other substrates, such as the lung-inflammation-related peptide des-Arg-bradykinin, also promote its cardiopulmonary protective effects [50]. Secondly, ADAM17 is a metalloproteinase which cleaves the extracellular domain of ACE2, releasing a soluble enzymatically active form of ACE2 into the plasma [51]. Future studies need to probe the role of soluble ACE2 and its regulation via post-translational modifications in the pathophysiology of PAH. Thirdly, the expression and function of ACE2 besides the pulmonary system, including the central nervous system and the gastrointestinal system, and its potentially protective effects against PAH should be investigated [52,53,54]. Furthermore, the interaction of ACE2 with other signaling pathways, such as the apelinergic system, need to be explored and therapeutically exploited [55]. Finally, the biomechanical aspects of PAH pathophysiology need to be studied in order to gain a better understanding of this disease and develop effective treatments. Specifically, we need to decipher pulmonary artery cell mechanosignaling in the setting of PAH and uncover its potential association with ACE2 signaling. Regarding the development of ACE2 signaling-related therapeutics, it is important for such drugs to be directly delivered to the cardiopulmonary system and act locally in order to be maximally effective. With this in mind, developing ACE2 pathway-modulating drugs that can be administered through inhalation may be a promising therapeutic strategy in PAH. Researchers should also compare well-established PAH therapies to agents targeting the ACE2/Ang-(1-7)/Mas 1 receptor signaling in order to appreciate the latter’s potential non-inferiority. Similarly, future studies should evaluate the combination of already approved drugs for PAH with drugs modulating the ACE2 signaling pathway in order to reveal potential synergistic effects. An interesting future study could be the assessment of the novel drug sotatercept (a TGF-β ligand trap), which is currently under clinical investigation, in combination with ACE2 or Mas 1 receptor activators. Delving deeper into the molecular underpinnings of ACE2 signaling in PAH will certainly provide the setting for developing effective drugs against this devastating disease.

## Figures and Tables

**Table 1:** Summary of ACE2 signaling-dependent molecular mechanisms involved in PAH pathophysiology. ACE2, angiotensin-converting enzyme 2; AMPK, AMP-activated protein kinase; Ang-(1-7), angiotensin-(1-7); ERK1/2, extracellular signal-regulated kinase 1/2; FAK, focal adhesion kinase; HIF-1, hypoxia-inducible factor 1; IL-6, interleukin 6; IL-1β, interleukin 1β; IL-10, interleukin 10; JAK2/STAT3, Janus kinase 2/signal transducer and activator of transcription 3; MDM2, murine double minute 2; NADPH, nicotinamide adenine dinucleotide phosphate; NO, nitric oxide; PA, pulmonary artery; PASMC, pulmonary artery smooth muscle cells; SMC, smooth muscle cell; TGF-β, transforming growth factor-β; TNF-α, tumor necrosis factor-α.

Effect	ACE2 Signaling-Dependent Molecular Mechanism	References
↓SMC proliferation and migration in PA	ERK1/2 and JAK/STAT3 signaling inhibition	[22]
↓Inflammation and oxidative stress	↓mRNA levels of proinflammatory cytokines (IL-6, IL-1β, TNF-α) and NADPH subunit (gp91phox), ↑anti-inflammatory cytokine IL-10	[21]
↓Fibrosis	↓mRNA levels of TGF-β and collagen fiber production	[21]
↑NO	ACE2-mediated phosphorylation (Ser1177) and dephosphorylation (Thr495) of endothelial nitric oxide synthetase	[24]
Cardiomyocyte hypertrophy inhibition	Ang-(1-7)-induced upregulation of a nitric oxide/guanosine 3′,5′-cyclic monophosphate-dependent pathway	[25]
Pulmonary vascular remodeling suppression	↑Apoptosis via Hippo signaling	[26]
↑PASMC proliferation and migration	↓ACE2 expression via HIF-1-dependent upregulation of microRNA let-7b	[29]
↑PASMC apoptosis	↓FAK signaling and ↑pro-apoptotic caspase-3	[30]
↑ACE2 protein stability and ↑Ang-(1-7) and NO levels	AMPK-related ACE2 phosphorylation at Ser680	[31]
↑ACE2 protein degradation	MDM2-mediated ACE2 ubiquitination at K788	[32]
↓ACE2 protein levels and activity	Autoantibodies against ACE2	[33,34]

## Data Availability

Data are contained within the article.
